# Cisplatin plus oral etoposide (EoP) combination is more effective than paclitaxel in patients with advanced breast cancer pretreated with anthracyclines: a randomised phase III trial of Turkish Oncology Group

**DOI:** 10.1038/sj.bjc.6602388

**Published:** 2005-02-22

**Authors:** F Icli, H Akbulut, A Uner, B Yalcin, E Baltali, M Altinbas, Ş Coşkun, S Komurcu, M Erkisi, A Demirkazik, F C Senler, O Sencan, A Büyükcelik, C Boruban, H Onur, N Zengin, S D Sak

**Affiliations:** 1 Department of Medical Oncology, Ibni Sina Hospital, Ankara University School of Medicine, Ankara 06100, Turkey; 2 Department of Medical Oncology, Gazi University Faculty of Medicine, Ankara, Turkey; 3 Department of Medical Oncology, Hacettepe University Faculty of Medicine, Ankara, Turkey; 4 Department of Medical Oncology, Erciyes University Faculty of Medicine, Kayseri, Turkey; 5 Department of Medical Oncology, GATA, Ankara, Turkey; 6 Department of Medical Oncology, Cukurova University Faculty of Medicine, Adana, Turkey; 7 Department of Pathology, Ankara University School of Medicine, Ankara, Turkey

**Keywords:** advanced breast cancer, cisplatin, etoposide, paclitaxel, anthracycline

## Abstract

Our objective was to determine whether oral etoposide and cisplatin combination (EoP) is superior to paclitaxel in the treatment of advanced breast cancer (ABC) patients pretreated with anthracyclines. From December 1997 to August 2003, 201 patients were randomised, 100 to EoP and 101 to paclitaxel arms. Four patients in each arm were ineligible. The doses of etoposide and cisplatin were 50 mg p.o. twice a day for 7 days and 70 mg m^−2^ intravenously (i.v.) on day 1, respectively, and it was 175 mg m^−2^ on day 1 for paclitaxel. Both treatments were repeated every 3 weeks. A median of four cycles of study treatment was given in both arms. The response rate obtained in the EoP arm was significantly higher (36.3 *vs* 22.2%; *P*=0.038). Median response duration was longer for the EoP arm (7 *vs* 4 months) (*P*=0.132). Also, time to progression was significantly in favour of the EoP arm (5.5 *vs* 3.9 months; *P*=0.003). Median overall survival was again significantly longer in the EoP arm (14 *vs* 9.5 months; *P*=0.039). Toxicity profile of both groups was similar. Two patients in each arm were lost due to febrile neutropenia. The observed activity and acceptable toxicity of EoP endorses the employment of this combination in the treatment of ABC following anthracyclines.

Survival in breast cancer is improving, mostly related to early diagnosis. However, at least 40% of the early cases present with recurrent disease following curative surgery±adjuvant systemic therapy. Although hormonal treatment is preferred for advanced breast cancer (ABC), chemotherapy (CT) is required for hormone receptor-negative and hormone refractory disease.

Anthracycline combinations have been commonly used to treat for ABC for a long time. Recently, taxanes became the standard treatment for these patients following anthracycline failure. In randomised trials, docetaxel (Taxotere®) was found comparable to 5-fluorouracil+vinorelbine (FUN) and more active than mitomycin C+vinblastine (MV), and methotrexate+5-fluororacil (MF) in this group of patients (Nabholtz *et al*, 1999; Sjöström *et al*, 1999; Bonneterre *et al*, 2002). However, there are limited numbers of randomised trials comparing the efficacy of paclitaxel to other drugs or combination CTs in anthracycline-pretreated patients. In a small phase II randomised trial, capecitabine was found to be as effective as paclitaxel in patients with ABC pretreated with anthracyclines (Talbot *et al*, 2002). Recently, given as a single agent, docetaxel was reported to be more active than paclitaxel in terms of overall response rates, time to progression (TTP) and survival in patients with ABC pretreated with anthracyclines (Ravdin *et al*, 2003).

Another active but less popular treatment in anthracycline-pretreated patients with ABC is etoposide+cisplatin (EoP). A higher response rate than that achieved with CMF was observed by giving EP as first-line treatment in a small randomised trial (Cocconi *et al*, 1991). Etoposide+cisplatin was also found as an active treatment for ABC following anthracyclines in the several phase II trials (Cox *et al*, 1989; Krook *et al*, 1990; Icli *et al*, 1992; Remick *et al*, 1996). Furthermore, 42 and 50% response rates with acceptable side effects were reported by prolonged administration of oral EoP in two phase II trials, which were higher than those obtained by giving paclitaxel in ABC patients pretreated with anthracyclines (Icli and Demirkazik, 1998; Fried *et al*, 2000). Cost of the drugs in EoP is much lower than taxanes.

With the premise that EoP may be more effective than paclitaxel, Turkish Oncology Group (TOG) decided to compare the efficacy of EoP with paclitaxel in anthracyline-pretreated patients with ABC in a phase III randomised trial.

## PATIENTS AND METHODS

### Patients

After obtaining written informed consent, patients with histologically or cytologically confirmed locally advanced or metastatic breast cancer were randomised if they meet the following criteria: measurable or evaluable disease (metastases to skin, pleura and peritoneum), age 18–70 years, performance status 2 or less according to WHO criteria, pretreatment with anthracyclines, negative hormone receptors or hormone refractory disease, adequate bone marrow reserve measured as neutrophil count ⩾2 × 10^9^ l^−1^ and platelet count ⩾100 × 10^9^ l^−1^, normal BUN, serum creatinine and bilirubin levels and AST and ALT levels <4 times upper normal limits.

Criteria for exclusion were presence of second primary malignancy or brain metastasis as the only metastatic site. Brain metastasis well controlled with radiotherapy, in addition to other sites of metastasis was not an exclusion criterion.

Patients with disease progression while receiving anthracycline-based CT for ABC, relapse within 6 months following adjuvant anthracycline-based CT and no response after two or more cycles of anthracycline-based CT for ABC, or responded to anthracyclines for ABC or received adjuvant anthracyclines and relapsed after 6 months (total dosage ⩾360 mg of doxorubicin or ⩾450 mg of epirubicin) were regarded as anthracycline pretreated.

### Study design

This was a prospective randomised nonblinded multicentre phase III study. No stratification was carried out for prognostic factors or centers. Patients were centrally randomised to either paclitaxel or EoP arms. The primary end point was TTP. Secondary end points were tumor response rate, duration of response and overall survival (OS).

### Treatment

Chemotherapy doses and schedules were as follows. Paclitaxel 175 mg m^−2^ intravenously (i.v.) on day 1 or etoposide 50 mg b.i.d p.o. daily for 7 days+cisplatin 70 mg m^−2^ i.v. on day 1 with adequate i.v. hydration every 3 weeks. At least two cycles of study treatment was planned for each patient, unless there was clear evidence of progression following the first cycle. Crossover was allowed for patients with progressive disease at any time. Also, patients with stable disease after at least two cycles of study treatment could be crossed over at the discretion of the investigator. Crossover was not mandatory.

Paclitaxel dose was reduced to 135 mg m^−2^ in case of previous RT to pelvis and vertebrae or if ALT and/or AST were more than three times upper normal limits. If grade 3–4 hematological toxicity was observed in the prior cycle of the treatment, paclitaxel was reduced to 135 mg m^−2^ (or 110 mg m^−2^ if prior dose was 135 mg m^−2^). Likewise, etoposide was reduced to 50 mg p.o. twice a day for 5 days and cisplatin to 50 mg m^−2^. Treatment was delayed if there was grade 2 or more toxicity at the scheduled date of study treatment.

### Assessment and follow-up

Physical examination, complete blood count, liver and renal function tests, serum CA-15-3, chest X-ray, abdominal ultrasonography (USG) or computerised tomoraphy were carried out before the first cycle of study treatment. Thorax computerised tomography (CT) was carried out only if lung metastasis was suspected from the chest X-ray. Physical examination and routine blood tests were repeated before each cycle of CT. Chest X-ray or thorax CT was required every 6 weeks during the treatment and every 3 months post-treatment. Likewise, abdominal USG/CT was required every 6 weeks during the treatment and every 3 months post-treatment if intra-abdominal metastasis was present. Response to treatment was evaluated every 6 weeks, unless there was evidence of progressive disease on physical examination in 3 weeks. The same assessment method used to determine the disease status at baseline was used consistently for efficacy evaluation throughout the study and follow-up. Routine post-treatment follow-up was every 1–3 months at the discretion of the investigator in case of lack of any symptoms or signs suggesting progressive disease.

Response to study treatment was assessed according to WHO criteria. Response rates were evaluated for the actual treatments patients had received. Responses were reviewed by two independent experts to confirm the response status blindly for treatment received. Response duration was measured from the date of response to date of progression. Time to progression was the duration between the first day of study treatment and date of progression. Likewise OS was accepted as the time interval between the first day of study treatment and date of death. Overall survival was calculated on intent-to-treat basis.

### Statistical considerations

A total of 146 events were planned based on the log-rank test, for a median TTP ratio of 1.65, for two-sided 0.05 type 1 error rates and 0.80 power. Comparisons between the response rates, patient characteristics and adverse events were carried out by using *χ*^2^ test. Time to progression and OS parameters were analysed using the Kaplan–Meier method and log-rank test. Stepwise Cox's regression analysis was used to assess the significant predictors for survival. Time interval from diagnosis and relapse to study treatment, CT, relative dose intensity (RDI), number of metastatic sites and age were used as continuous variables and type of study treatment as a dichotomous variable.

## RESULTS

### Patient characteristics

Between December 1997 and August 2002, 201 patients from seven Oncology centres in Turkey were enrolled. A total of 100 patients were randomised to EoP arm and 101 to paclitaxel arm. Randomisation was carried out centrally by the data centre of TOG. Four patients in each arm were ineligible because one patient in each arm had poor performance status and were lost before the start of study treatment, three patients in the EoP and two in the paclitaxel arms withdrew their consents and one patient in the paclitaxel arm was injured in a car accident and the treatment could never be started. Thus, there were 96 eligible patients in the EoP arm and 97 in the paclitaxel arm. Patient characteristics including prior treatments are depicted in Table 1. There were no substantial differences between the two arms. There were two evaluable patients, one in paclitaxel and one in EoP arms, who had cytologically proven metastatic disease of pleura with effusion. All other patients had measurable disease. The median number of treatment cycles were 4 (ranges 1–8) for both arms. In total, 68 patients in the EoP and 75 patients in the paclitaxel arm received three to six cycles of treatment. Only four and seven patients in the EoP and paclitaxel arms, respectively, were given one cycle of treatment. Relative dose intensities were 85.17 and 85.74% for the etoposide and cisplatin, respectively, *vs* 89.27% for the paclitaxel. While paclitaxel dosage was reduced in five patients (5%), EP was reduced in 10 patients (10%) because of previous radiation to pelvis.

### Efficacy

A total of 91 patients in the EoP arm and 95 in the paclitaxel arm were evaluable for response. One patient was given paclitaxel instead of assigned EoP and was excluded from response evaluation. However, her survival duration was included in the EoP arm on intent-to-treat basis. Two patients in the EoP arm and three in the paclitaxel arm died before any response evaluation. Likewise, two patients in the EoP arm did not come for further treatment following the first cycle. Response to study treatment is shown in Table 2. Total response rates were 36.3 and 22.2% in the EoP and paclitaxel arms, respectively (*P*=0.038). Complete response was achieved in three patients in each arm.

Five out of 42 patients (11.9%) crossed over to EoP from paclitaxel *vs* two out of 30 patients (6.7%) crossed over to paclitaxel achieved a PR. Until July 2003, disease progression was observed in 182 patients and 165 had died.

**Figure 1:**
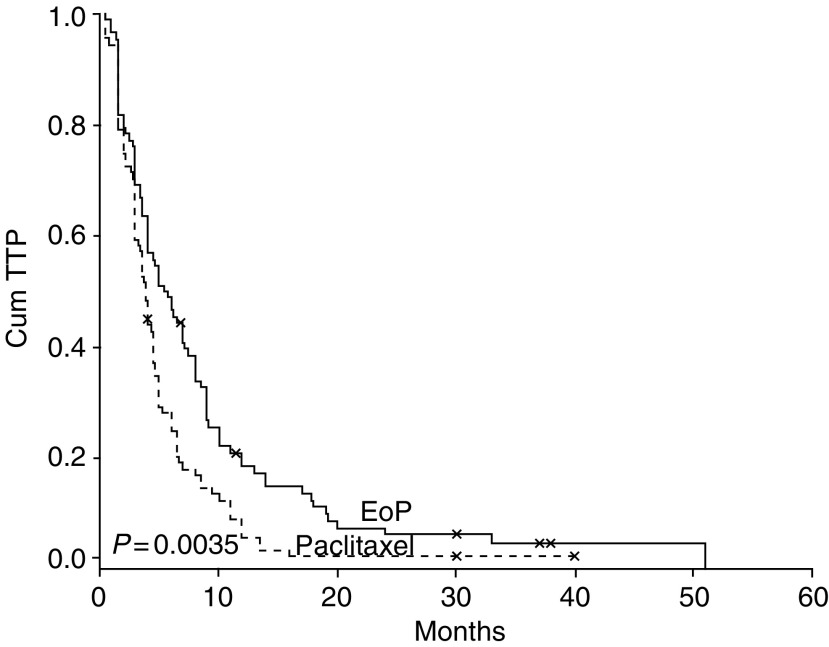
Kaplan–Meier curves for TTP according to the assigned arms.

**Figure 2:**
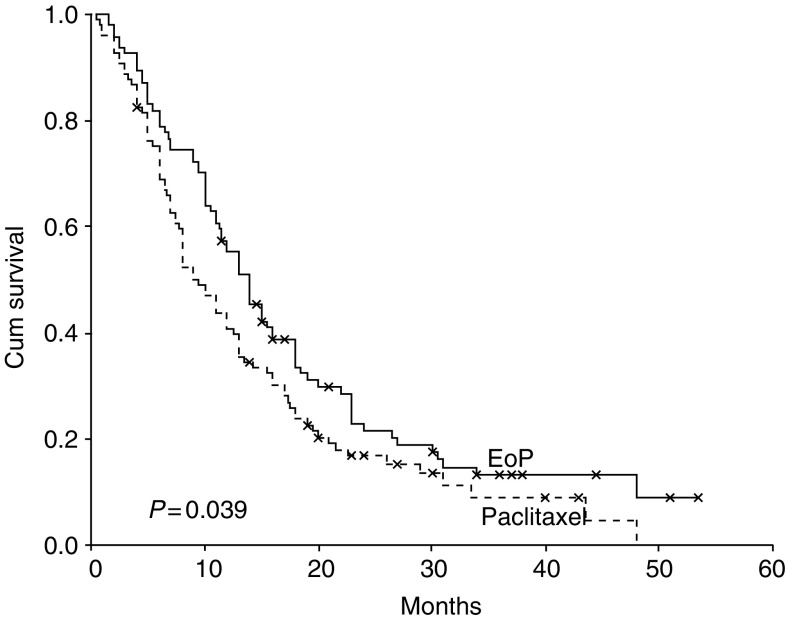
Kaplan–Meier curves for OS according to the assigned arms.

The duration of response was not significantly different between the two arms (Table 2). Median response duration for patients in the EoP and paclitaxel arms was 7 and 4 months, respectively (*P*: 0.132). Time to progression was significantly in favour of the EoP arm (*P*: 0.003). Median TTP was 5.5 months for the EoP arm and 3.9 months for the paclitaxel arm (Figure 1). Likewise, median survival for patients in the EoP arm (14 month) was longer than those in the paclitaxel arm (9.5 months) (*P*=0.039) (Figure 2). The 1-year survival rate was also in favour of the EoP arm, although the difference was not statistically significant (55.3 *vs* 40.7%; *P*=0.168).

Although not significant, overall response rates were in favour of EoP arm in patients who had adjuvant anthracyclines more than 6 months ago and those who were resistant to prior anthracyclines for metastatic disease or relapsed within 6 months of adjuvant anthracyclines when compared to paclitaxel arm (36.8 *vs* 33.3% and 23.3 *vs* 18.8%, respectively). In patients who responded to prior anthracycline treatment, EoP has yielded a significantly higher response rate (55.6 *vs* 17.9%; *P*=0.005). While there was no significant difference in terms of TTP in patients with adjuvant anthracyclines more than 6 months ago (5.0±1.0 *vs* 4.6±0.5 months; *P*=0.611), it was significantly higher in favour of EoP arm in anthracycline-responsive or -resistant patients (4.5±1.1 *vs* 3.0±1.1 months, *P*=0.005; 6.0±1.0 *vs* 3.5±0.3 months, *P*=0.006).

Multivariate analysis including time interval from diagnosis and relapse to study treatment, CT, RDI, number of metastatic sites, age and type of study treatment showed that only type of study treatment had significant impact on survival (*P*=0.0281). The only other parameter that was found to have an impact on OS in multivariate analysis close to statistical significance was the number of metastatic sites (*P*=0.059).

### Toxicity

Chemotherapy toxicity is shown in Table 3. Complete blood counts were required every 3 weeks unless a febrile episode occurred. Therefore, the nadirs for neutrophils and platelets between the treatment cycles were not assessed adequately. Grade 4 haematologic toxicities were observed in four and six patients in the EP and paclitaxel arms, respectively. Delay of study treatment for at least 1 week due to myelosuppression occurred in 21 patients in the EoP group *vs* in three patients in the paclitaxel group. Likewise, 23 and 17 patients had delayed treatments from other causes in the EoP and paclitaxel groups, respectively. Two patients in each arm were lost probably related to treatment toxicity. The causes of deaths were febrile neutropenia. Also, one patient was lost with fulminant hepatitis 4 weeks after the first dose of paclitaxel. Grade 3 nausea was more common in the EoP arm (15 patients). On the other hand, there were few patients with grade 3 arthralgia and neurologic toxicity in the paclitaxel arm. Likewise, three patients in the paclitaxel arm suffered from congestive heart failure while taking study treatment.

## DISCUSSION

This is the first randomised trial comparing the efficacy of paclitaxel with other CT in anthracycline-pretreated patients with breast cancer. In this trial, EoP was found to have higher efficacy in terms of response rates, TTP and OS when compared to paclitaxel.

The additive and synergistic effects of cisplatin and etoposide in experimental models have been reported previously (Burchenal *et al*, 1979; Mabel and Little, 1981). Although EP has been commonly employed in lung cancer and germ cell tumors, it is not a well-established treatment for patients with breast cancer. Nevertheless, cisplatin was found to be an active drug in breast cancer in 1980s (Sledge and Roth, 1989). However, early trials with single agent i.v. etoposide in previously treated patients with ABC were not promising (Sledge, 1991).

The efficacy of EP was assessed in eight phase 2 trials including 260 patients previously treated for ABC (Athanassiades *et al*, 1986; Cocconi *et al*, 1986; Giaccone *et al*, 1988; Cox *et al*, 1989; Krook *et al*, 1990; Icli *et al*, 1992; Ceci *et al*, 1995; Remick *et al*, 1996). A total response rate of 26.8% was obtained by giving etoposide 100–130 mg m^−2^ i.v. for 3–5 days and cisplatin 60–100 mg m^−2^ i.v. every 3 weeks to these heavily pretreated patients. The highest rate of grade 3–4 leukopenia was 31% in one trial, and altogether four toxic deaths were reported in these trials.

Following the emergence of oral etoposide, the role of prolonged oral etoposide in the treatment of breast cancer was investigated in five phase II trials (Calvert *et al*, 1993; Martin *et al*, 1994; Palombo *et al*, 1994; Atienza *et al*, 1995; Bontenbal *et al*, 1995). Unlike the results of single agent i.v. etoposide, the overall response rate was 23.8% in 143 patients with ABC, most of whom were pretreated. Etoposide was utilised 50–100 mg p.o. for 14–21 days every 3–4 weeks in these trials. Myelosuppression, more prominent with 21 days of etoposide, and alopecia were notable toxicities in these trials.

So far, only two phase II trials looked into the role of oral EoP in ABC (Icli and Demirkazik, 1998; Fried *et al*, 2000). In our phase II trial, we have used the same dosage and schedule of EoP as in the present study. Out of 35 (42.8%) heavily pretreated patients, 15 responded. Median response duration and OS were 6 and 8 months, respectively. Grade 3 leukopenia was observed in 14.3% of the patients and only one patient had grade 4 anaemia.

A lower dosage of cisplatin (50 mg m^−2^) and longer duration of oral etoposide (50 mg m^−2^ for 17 days) were utilised in the second trial by Fried *et al*. In 26 patients previously exposed to anthracyclines, 50% response rate with 7 months of response duration has been reported (Fried *et al*, 2000). Four patients (15%) required hospitalisation for neutropenic fever in that trial. The response rate achieved in the present randomised study (36.3%) is close to that obtained in our previous phase 2 trial (42.8%). Likewise, the 22.2% response rate obtained in the paclitaxel arm is comparable with those achieved in previous trials employing 175 mg m^−2^ i.v. paclitaxel every 3 weeks in anthracycline-pretreated patients (Abrams *et al*, 1995). Both results of the past phase 2 trials and present randomised trial are in favour of EoP when compared to paclitaxel. On the other hand, myelotoxicity of EoP was somewhat higher than that of paclitaxel (18 *vs* 11% grade 3–4 toxicity). Likewise, more patients in the EoP arm had delayed treatment interval due to toxicity. One might argue that the efficacy of paclitaxel could be increased by employing higher and more myelotoxic dosages. However, a randomised trial failed to show any favourable effect of higher than 175 mg m^−2^ of paclitaxel every 3 weeks in ABC, which excludes such an explanation of the lower efficacy of paclitaxel when compared to EoP in our trial (Winer *et al*, 2004).

Also, it was gratifying to see that both TTP and OS were significantly higher in the EoP arm when compared to paclitaxel arm, which has not been usual for randomised CT trials involving patients with ABC. Both TTP and OS curves show a stable progress in favour of the EoP arm (Figures 1 and 2). Although there were some minor differences in terms of patient characteristics between the two groups, they had no significant impact on TTP and OS in multivariate analysis. Moreover, there were no notable differences between the groups in terms of prior treatments and setting of study treatments, which rule out the role of these factors on the favourable results of EoP arm.

The myelotoxicity, however, was higher in the EoP arm when compared to T. In all, 21 patients in the EoP arm *vs* only three patients in the paclitaxel arm had at least 7 days of treatment delays due to myelosuppression. Likewise, nausea and asthenia were more common in the EoP arm. Probably more myelotoxicity in each arm would be noted if CBCs were repeated weekly instead of every 3 weeks. Two deaths in each arm following febrile neutropenia also suggest that the grade 4 neutropenia was more common than noticed for both EoP and paclitaxel arms.

Toxicities observed in several phase II as well as in two randomised trials assessing the efficacy of EP in breast cancer have limited the use of this combination in breast cancer (Giaccone *et al*, 1988; Krook *et al*, 1990; Cocconi *et al*, 1991; Remick *et al*, 1996; Icli *et al*, 2001). However, the dosage of cisplatin in all these trials was higher when compared to the present trial (100 *vs* 70 mg m^−2^). A randomised phase 2 study comparing low (60 mg m^−2^) *vs* high (100 mg m^−2^) doses of cisplatin in the EP combination against breast cancer concluded that the dose of cisplatin had no significant effect in terms of TTP and OS (Ceci *et al*, 1995). Also, the dose intensity of both cisplatin and etoposide in the present trial was found to have no significant impact on both TTP and OAS in the multivariate analysis. Therefore, it may be premised that similar efficacy could be achieved by lower and less toxic dosages of both cisplatin and oral etoposide in ABC.

In the 1990s, paclitaxel 175 mg m^−2^ i.v. every 3 weeks was considered as the treatment of choice following anthracyclines for patients with ABC (Nabholtz *et al*, 1996). Our randomised trial of EoP *vs* paclitaxel proves that EoP is more active in this group of patients. Significantly improved survival in the EoP arm in this trial is a rarely observed phenomenon in randomised trials of ABC treatment. Approximately 10 times lower price of EoP than paclitaxel also favours this treatment, especially in countries with limited sources of health expenditure. However, we should admit that absence of quality of life assessment is the weaknesses of the present trial.

In conclusion, results obtained in the present trial supports the use of EoP in the treatment of ABC. Further randomised trials will enlighten the efficacy of this relatively old treatment when compared to the new active drugs in breast cancer. Likewise, it will be interesting to see the efficacy of this treatment combined with herceptin in HER2-positive patients.

## Figures and Tables

**Table 1:** Patient characteristics

	**EoP (*n*=96)**	**Paclitaxel (*n*=97)**	***P*-value**
Median age (years) (range)	47 (26–69)	49 (24–70)	0.377
*Performance status (WHO)*			
0	20	17	
1	50	45	0.401
2	26	35	

*Site of metastasis*			
Locally advanced	4	3	
Skin	47	37	0.148
Lymph node	22	15	
Lung	47	47	
Liver	35	46	0.145
Bone	45	39	0.385
Brain	3	3	
Peritoneum	5	2	

*Number of metastatic sites*			
1	29	29	
2	33	44	0.354
3 or more	34	24	

*Hormone receptor*			
ER/PR+	29	31	
ER/PR−	15	22	0.298
Unknown	52	44	

*Oncogene expression*			
HER2+	11	11	
HER2−	9	13	0.763
Unknown	76	73	
TIDTR	28.4±5.0	28.9±3.9	0.350

*Prior treatments*			
Surgery	72	72	1.000
Radiotherapy			0.285
Adjuvant	17	17	
Metastatic	18	11	
Hormone			0.158
Adjuvant	10	18	
Metastatic	14	16	

*Prior anthracyclines* a			0.663
1	33	32	
2	2	4	
3	18	13	
4	43	48	

*Setting of study drugs*			0.947
First line	18	20	
Second line	59	58	
Third line	19	19	

EoP: cisplation+oral etoposide; WHO=World Health Organization; TIDTR: time from initial diagnosis to treatment.

a 1: disease progression while receiving anthracycline-based CT for ABC; 2: relapse within 6 months following adjuvant anthracycline-based CT; 3: no response after two or more cycles of anthracycline-based CT for ABC; 4: responded to anthracyclines for ABC or received adjuvant anthracyclines and relapsed after 6 months (total dosage ⩾360 mg of doxorubicin or ⩾450 mg of epirubicin). ER=oestrogen receptor; PR=progesterone receptor; HER2=c-ErbB-2.

**Table 2:** Results of the response evaluation and time-related variables

	**EoP (*n*=91)**	**T (*n*=94)**	***P*-value**
*Response type* (%)			
OR	33 (36.3)	21 (22.3)	0.038
CR	3 (3.3)	3 (3.2)	
PR	30 (33.0)	18 (19.1)	
Stable	44 (48.3)	53 (56.4)	
Progression	14 (15.4)	20 (21.3)	


	**Paclitaxel × EoP: 42 patients**	**EoP × paclitaxel: 30 patients**	
*Crossover*			
PR	5 (11.9)	2 (6.7)	

Response duration (95% CI)	7.0±1.1 (4.9–9.1)	4.0±0.5 (3.0–4.9)	0.132
TTP (95% CI)	5.5±0.9 (3.7–7.3)	3.9±0.3 (3.4–4.4)	0.0035
OS (95% CI)	14.0±1.2 (11.7–16.3)	9.5±1.0 (7.4–11.5)	0.039
1-Year survival rate (%)	55.3±5.1	40.7±5.0	0.048

EoP=etoposideplus cisplatin; TTP=time to progression; CI=confidence interval. OR=overall response; CR=complete response; PR=partial response.

**Table 3:** Summary of common toxicities

	**EoP (*n*=96)**		**T (*n*=97)**	
	**Grade 3**	**Grade 4**	**Grade 3**	**Grade 4**
*Haematological*				
Anaemia	3	0	0	0
Neutropenia	14	4^a^	5	6^b^
Thrombocytopenia	1	0	1	1

Nausea	15	0	1	0
Arthralgia	0	0	3	0
Neurologic	0	0	1	0
Cardiac	1^c^	0	3^d^	0
Toxic death		2		3

a One patient had febrile neutropenia.

b Two patients had febrile neutropenia.

c Supraventricular tachycardia.

d Congestive heart failure.
